# Repeat Diagnoses of Bethesda Category III Thyroid Nodules: What To Do Next?

**DOI:** 10.1371/journal.pone.0130138

**Published:** 2015-06-26

**Authors:** Mi Ri Yoo, Hye Mi Gweon, Ah Young Park, Kyung Eun Cho, Jeong-Ah Kim, Ji Hyun Youk, Eun Ju Son

**Affiliations:** Department of Radiology and Research Institute of Radiological Science, Yonsei University College of Medicine, Gangnam Severance Hospital, Seoul, Republic of Korea; The Ohio State University, UNITED STATES

## Abstract

**Objectives:**

To assess the malignancy rates of thyroid nodules repeatedly classified as Bethesda category III on fine needle aspiration (FNA), and to suggest management guidelines for these lesions.

**Methods:**

This is a retrospective study that included 395 thyroid nodules categorized as Bethesda III undergone either surgery or ultrasound (US) follow-up. There were 67 nodules classified a second time as Bethesda category III on repeat FNA. We compared malignancy rates, clinicopathologic and ultrasonographic characteristics between direct surgery and repeat FNA groups and between the initial and repeat Bethesda category III groups, each. And in the repeat Bethesda III group, clinicopathologic and US variables were compared between benign and malignant nodules.

**Results:**

Incidence of concurrent cancer, underlying thyroiditis and positive BRAF mutation were significantly higher in 142 nodules with direct surgery than 243 nodules with repeat FNA (*p* < 0.05). Of the 395 nodules with Bethesda category III cytology on initial FNA, the malignancy rate was 59.5%. In 67 nodules with repeat Bethesda III classification, however, the malignancy rate was 73.1% (*p* < 0.05). However, none of the variables were significantly different between the initial Bethesda category III group and the repeat Bethesda category III group (*p* > 0.05). In the repeat Bethesda category III group, solid consistency, irregular/microlobulated margins, nonparallel shape, and number of suspicious findings or “suspicious malignant” US assessments were associated with a high malignancy rate (*p* < 0.05). On multivariate logistic regression analysis, the factor associated with malignancy in the repeat Bethesda category III group was irregular/microlobulated margin (odds ratio = 15.576; 95% CI, 2.097–115.6804, *p* = 0.007) with a sensitivity, specificity, positive and negative predictive values, and accuracy of 81.6%, 83.3%, 93.0%, 62.5% and 82.1%, respectively.

**Conclusion:**

Thyroid nodules with repeated Bethesda category III classification and irregular/microlobulated margins on US are at increased risk of malignancy, and operative management should be considered as opposed to repeat FNA.

## Introduction

The Bethesda System for Reporting Thyroid Cytopathology has standardized thyroid fine needle aspiration (FNA) results and has facilitated effective communication among clinicians [1]. However, the Bethesda category III classification, atypia of undetermined significance/follicular lesion of undetermined significance (AUS/FLUS) has remained ambiguous with respect to risk of malignancy and guidelines for management [1–3]. Although the predicted risk of malignancy for Bethesda category III nodules ranges from 5–15% and the most frequently recommended management is repeat FNA after 3 months, recent studies have shown a higher risk of malignancy and higher rates of immediate surgery [4,5]. Consequently, many studies have examined ways to better predict malignancy in nodules with Bethesda category III classification on FNA according to clinical data [5–8], laboratory data [6], ultrasonographic findings [5], and nodule size [5–8].

Although previous studies have suggested a method for management of Bethesda III nodules, repeat FNA is still frequently performed. In a recent prospective study, 48.6% of initial Bethesda category III nodules persisted as category III on repeat FNA [9]. These repeat category III nodules can be problematic because of increased patient anxiety, cost, and delayed definitive diagnosis. Few studies have looked at repeat Bethesda category III nodules, and there is currently no recommended guideline for their management. Therefore, the aim of our study was to evaluate the clinicopathologic features of repeat Bethesda category III nodules on FNA, to determine clinical and ultrasonographic predictors of malignancy, and to suggest management guidelines for these nodules.

## Materials and Methods

The institutional review board of Gangnam Severance hospital approved of this retrospective observational study and required neither patient approval nor informed consent for our review of patients’ images and records. However, written informed consent was obtained from all patients for US-guided FNA prior to each procedure as a daily practice.

### Patient population

At our institution, US-FNAs were performed on either a thyroid nodule larger than 3mm in diameter with suspicious US features or the largest thyroid nodule if no suspicious US features were detected. Because our institution is a referral center, patients referred from outside clinics for US-FNA are indicated for aspiration.

From January 2010 to December 2012, 11988 thyroid nodules were undergone US-guided FNA at our institution, which is a tertiary referral center. The initial aspirates from 772 nodules (6.4%) were reported as Bethesda category III. Among them, 377 nodules were excluded for a lack of adequate follow-up of at least 1 year. In this study, included were remained 395 nodules with surgically confirmed histopathology or clinical follow up for more than 1 year. Clinically benign nodule was defined as thyroid nodule that had been resolved on follow-up US or was Bethesda category II on repeat FNA unchanged or decreased in size over the course of one year [3,5,10].

Clinical features, ultrasonographic findings, and cytopathologic records were reviewed for each case, retrospectively. Our standard approach to an initial Bethesda III diagnosis has been a repeated FNA. Surgical resection was recommended for the patients with concurrent cancer, positive BRAF mutation, compressive symptoms, cosmetic issues due to large goiter, clinical suspicion, and preference by patients or physicians, whereas patients with a benign diagnosis (Bethesda II) on repeat FNA were followed up. However, variable management approaches were applied in individual cases based on an overall assessment of clinical characteristics as noted above.

### Image analysis

US and FNA were performed with 7- to 15-MHz (HDI 5000; Philips Medical Systems, Bothell, WA) and 5- to 12-MHz linear array transducers (iU22; Philips Medical Systems). Real-time US examinations were performed by one of six radiologists with 5–16 years of experience in thyroid imaging. A retrospective analysis was performed for the US findings of internal components, echogenicity, margins, calcifications, shape, and final assessment of the nodules by two radiologists in consensus (MRY and EJS with 5 and 13 years of experience in thyroid US imaging, respectively), who were blinded to the clinicopathologic data. These data were entered into a database using a computerized spreadsheet (Excel; Microsoft. Redmond, WA). Internal components were divided into solid nodules, mixed nodules, and cysts. Echogenicity was classified as hyperechoic, isoechoic, hypoechoic, or markedly hypoechoic. For mixed solid and cystic nodules, the echogenicity of the solid components was used for classification. Margins were classified as circumscribed, microlobulated, or irregular. Calcifications were divided into microcalcifications, macrocalcifications, eggshell calcifications, and no calcifications. Shape was designated as either parallel or nonparallel. On the basis of these US features, those thought to be associated with malignancy were solid, had marked hypoechogenicity, microlobulated or irregular margins, microcalcifications, and nonparallel shape based on published criteria [5,11]. Final assessment of the thyroid nodules determined them to be ‘‘probably benign” when none of the suspicious US features described were present, and ‘‘suspicious malignant” when a thyroid nodule showed one or more of the malignant features described previously. The total number of suspicious ultrasonographic features present in each thyroid nodule was counted and recorded for analysis.

### US-guided FNA and cytologic analysis

US-guided FNA was performed by the same radiologist who performed the real-time US examinations. US-guided FNA was performed with a 23 gauge needle attached to a 2 mL disposable syringe without using an aspirator. Two alcohol-fixed smears underwent Papanicolaou staining, while the remainder was prepared for Thin Prep by rinsing the needle in Cytolyt (Hologic, Marlborough, MA). On-site evaluation was not performed routinely. All cases were reported by one cytopathologist using a six-tiered diagnostic system with the Bethesda System for Reporting Thyroid Cytopathology [1]. Cytologic diagnoses were made as follows: (1) nondiagnostic or unsatisfactory (Bethesda system I), (2) benign (Bethesda system II), (3) AUS/FLUS (Bethesda system III), (4) follicular neoplasm or suspicious for a follicular neoplasm (Bethesda system IV), (5) suspicious for malignancy (Bethesda system V), and (6) malignant (Bethesda system VI). The cytologic results were unsatisfactory when less than six clusters of thyroid follicular cells containing no identifiable colloid were observed in a given preparation. Cases that were classified as Bethesda I were not included in this study.

The presence of a BRAF mutation was evaluated by polymerase chain reaction-restriction fragment length polymorphism (PCR-RFLP) or direct sequencing.

### Data and statistical analysis

Cytopathologic results from surgery and repeat FNA as well as clinical follow-up with US were used as a standard reference. Histopathologically confirmed malignancies were defined as malignant outcomes, and benign lesions on surgical histopathology or second FNA cytology without interval change or decreased in size of the mass over a one year period were classified as benign. Outcome classification was solely based on the diagnosis made with tissue from the targeted nodule.

We compared malignancy rates, clinicopathologic characteristics (sex, age, concurrent cancer, BRAF mutation or underlying thyroiditis), and ultrasonographic characteristics (lesion size, composition, echogenicity, margin, calcification, shape, final US assessment, and number of suspicious US findings) between direct surgery group and repeat FNA group and between the initial and repeat Bethesda category III groups, each. And we compared malignancy rate, sex, age and ultrasonographic characteristics between benign and malignant nodules in the repeat Bethesda category III group. Categorical variables and malignancy rate were compared using χ2 tests and Fisher’s exact tests. Continuous variables were compared using Student’s t-tests. Multivariate logistic regression analysis with a forward stepwise selection method and odds ratio was performed to select independent predictors of malignancy in the repeat Bethesda category III group.

Statistical analyses were performed using a commercial software program (SAS, version 9.1.3, SAS Institute Inc., Cary, NC). Differences between groups were considered to be statistically significant at a *p*-value < 0.05.

## Results

Total 395 Bethesda category III nodules from 388 patients were included in this study. Of these 388 patients, 314 were female and 74 were male. The mean age was 47.5± 11.5 (range 18–76) years. The mean size of the 395 thyroid nodules on US was 12.3±10.4mm (range 3–62 mm). A total of 287 nodules underwent surgical confirmation with or without repeat FNA, and 108 clinically benign nodules were followed up with US in this study. In 108 clinically benign nodules, 13 nodules had been resolved on follow-up US and 95 nodules were Bethesda category II on repeat FNA unchanged or decreased in size over the course of a year. The mean follow-up period for 95 clinically benign nodules was 15.4 months (range 12–44 months).

**Fig 1:**
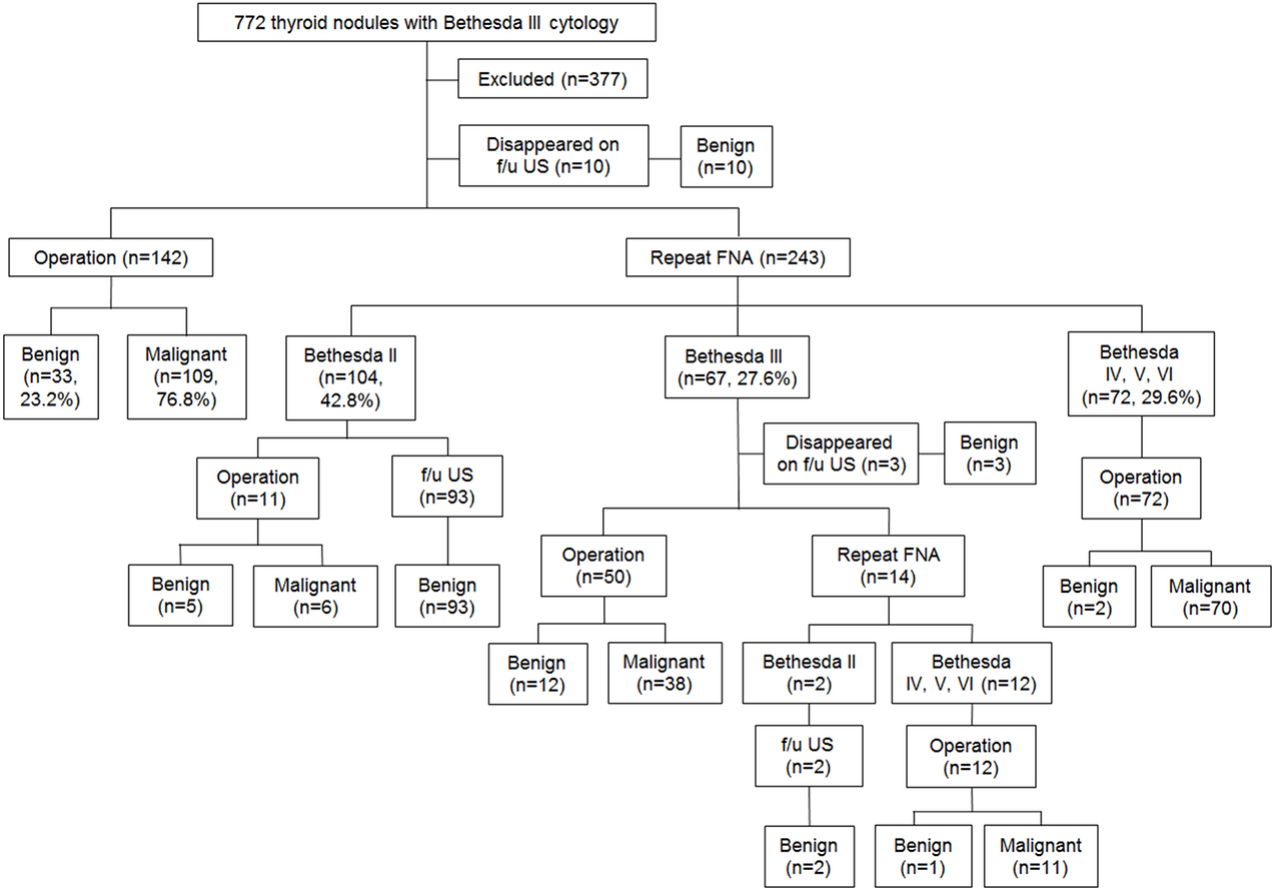
Diagnostic outcomes of 772 thyroid nodules with Bethesda category III cytology. f/u, follow-up; US, ultrasound; FNA, fine needle aspiration.

Among the 395 thyroid nodules classified as Bethesda category III on initial FNA, 234 (59.2%) were diagnosed as malignant on surgical histopathology. Of these 395 nodules, 142 (35.9%) underwent direct surgery and 243 (61.5%) underwent repeat FNA. When comparing clinicopathologic and US features between the direct surgery group and the repeat FNA group, incidence of concurrent cancer, underlying thyroiditis and positive BRAF mutation were significantly higher in direct surgery group (p < 0.05) (Table 1). Of the 243 nodules undergone second FNA, 42.8% (104/243) were Bethesda category II, 27.6% (67/243) were Bethesda III, and 29.6% (72/243) were Bethesda category IV, V, or VI (Fig 1).

**Table 1:** Comparison of Malignancy Rates, Clinical and Ultrasonographic Features between Direct Surgery and Repeat FNA Groups.

	Direct surgery	Repeat FNA	*p*-value
Malignant rate	109/142 (76.8%)^a^	126/243 (51.9%)	0.000
Age (years)^*^	47 ± 16	47 ± 11	0.712
Sex			1.000
Male	27/141(19.1%)^b^	45/239 (18.8%)	
Female	114/141 (80.9%)	194/239 (81.2%)	
Concurrent cancer	28/142 (19.7%)	5/243(2.1%)	0.000
Nodule size (mm)*	13.3 ± 11.8	11.8 ± 9.7	0.185
Composition (solid)	122/142 (85.9%)	204/243 (84.0%)	0.606
Echogenicity (marked hypoechogenicity)	29/142 (20.4%)	35/243 (14.4%)	0.126
Margin (irregular/microlobulated)	77/142 (54.2%)	128/243 (52.7%)	0.769
Calcification (microcalcification)	31/142 (21.8%)	44/243 (18.1%)	0.373
Shape (nonparallel)	53/142 (37.3%)	76/243 (31.3%)	0.225
Number of suspicious US findings*	2.2 ± 1.4	2.0 ± 1.3	0.187
Final US assessment (suspicious)	103/142 (72.5%)	167/243 (68.7%)	0.431
BRAF^c^	11/21 (52.4%)	10/62 (16.1%)	0.001
Underlying thyroiditis	24/142 (16.9%)	23/243 (9.5%)	0.032

*Numbers present the mean±standard deviation

^a^Number of nodules with suspicious features / total nodules

^b^Number of patients / total patients

^c^Positive BRAF mutation

Of those nodules that were classified as repeat Bethesda category III on the second FNA, 74.6% (50/67) underwent surgery and 20.9% (14/67) underwent a third FNA. The overall malignancy rate for the repeat Bethesda category III group was 73.1% (49/67), which was significantly higher than that of initial Bethesda category III group (59.5%) (*p* = 0.034). In comparing clinicopathologic and US features between the initial Bethesda category III group and the repeat Bethesda category III group, none of the variables were significantly different (*p* > 0.05) (Table 2).

**Table 2:** Comparison of Malignancy Rates, Clinical and Ultrasonographic Features between Initial and Repeat Bethesda Category III Groups.

	Initial Bethesda category III group	Repeat Bethesda category III	*p*-value
Malignant rate	235/395 (59.5%) ^a^	49/67 (73.1%)	0.034
Age (years)*	48 ± 12	47 ± 11	0.921
Sex			0.455
Male	74/388 (19.1%)^b^	10/67 (14.9%)	
Female	314/388 (80.9%)	57/67 (85.1%)	
Concurrent cancer	33/395 (8.4%)	4/67 (6.0%)	0.506
Nodule size (mm)*	12.3 ± 10.4	14.6 ± 11.6	0.127
Composition (solid)	336/395 (85.1%)	54/67 (80.6%)	0.351
Echogenicity (marked hypoechogenicity)	67/395 (17.0%)	10/67 (14.9%)	0.679
Margin (irregular/microlobulated)	212/395 (53.7%)	43/67 (64.2%)	0.110
Calcification (microcalcification)	76/395 (19.2%)	11/67 (16.4%)	0.585
Shape (nonparallel)	131/395 (33.2%)	26/67 (38.8%)	0.367
Number of suspicious US findings*	2.1 ± 1.3	2.3 ± 1.2	0.635
Final US assessment (suspicious)	278/395 (70.4%)	43/67 (64.2%)	0.707
BRAF^c^	21/85 (24.7%)	8/29 (27.6%)	0.758
Underlying thyroiditis	53/395 (13.4%)	10/67 (14.9%)	0.740

*Numbers present the mean±standard deviation

^a^Number of nodules with suspicious features / total nodules

^b^Number of patients / total patients

^c^Positive BRAF mutation

Of those nodules classified as Bethesda category II on the second FNA, 10.6% (11/104) underwent surgery. Those 11 thyroid nodules with Bethesda category II underwent surgical confirmation due to ≥2 suspicious US features (n = 7), concurrent cancer lesions (n = 2) and ≥ 45 mm in diameter (n = 2). Of the 11 Bethesda II lesions resected surgically, 6 were malignant, of which 5 had ≥3 suspicious US features and 1 was 45mm in diameter.

### Clinical and ultrasonographic features of repeat Bethesda category III nodules

The malignancy rates for each clinical and ultrasonographic feature in repeat Bethesda category III nodules are shown in Table 3. Solid, irregular/microlobulated margins, nonparallel shape, and “suspicious malignant” final US assessment were significantly associated with high malignancy rates (*p* < 0.05), and the number of suspicious findings identified in malignant nodules was significantly higher than in benign nodules (*p* < 0.05).

**Table 3:** Clinical and Ultrasonographic Characteristics of Repeat Bethesda Category III Nodules.

	Benign	Malignant	*p*-value
Age (years)*	50 ± 10	47 ± 11	0.314
Sex			0.808
Male	3/18 (16.7%) ^a^	7/49 (14.3%)	
Female	15/18 (83.3%)	42/49 (85.7%)	
Nodule size (mm)*	17.0 ± 13.7	13.7 ± 10.7	0.303
Composition (solid)	11/18 (61.1%)^b^	43/49 (87.8%)	0.032
Echogenicity (marked hypoechogenicity)	1/18 (5.6%)	9/49 (18.4%)	0.267
Margin (irregular/microlobulated)	3/18 (16.7%)	40/49 (81.6%)	0.000
Calcification (microcalcification)	1/18 (5.6%)	10/49 (20.4%)	0.264
Shape (nonparallel)	3/18 (16.7%)	23/49 (46.9%)	0.024
Number of suspicious US findings*	1.1 ± 1.0	2.6 ± 0.9	0.000
Final US assessment (suspicious)	4/18 (22.2%)	39/49 (79.6%)	0.000

*Numbers present the mean±standard deviation

Table 4 summarizes the results of the multivariate logistic regression analysis of clinical and ultrasonographic variables in the repeat Bethesda category III group, in which irregular/microlobulated margins (odds ratio, 15.576; 95% CI: 2.097, 115.680; *p =* .007) was an independent predictive factor of malignancy in women with repeat Bethesda category III nodules.

**Table 4:** Multivariate Analysis of Clinical and Ultrasonographic Characteristics for Predicting Malignancy of Repeat Bethesda Category III Nodules.

	OR (95% CI)	*p*-value
Composition (solid)	2.157 (0.207–22.447)	0.520
Margin (irregular/microlobulated)	15.576 (2.097–115.680)	0.007
Shape (nonparallel)	4.070 (0.510–32.446)	0.185
Final US assessment (suspicious)	1.781 (0.161–19.745)	0.638

OR, odds ratio; CI, confidence interval

## Discussion

The percentage of thyroid nodules assigned as Bethesda category III on the initial FNA was 6.4% and the risk of malignancy was 59.5% in this study. The malignancy rate was relatively high compared to the proposed malignancy rate of 5–15% in the Bethesda system. However, many recent studies also reported higher rate of malignancy than proposed one. The risk of malignancy for patients undergoing surgery with Bethesda category III nodules has been reported to be 37.8–55.5% [4,5,12]. Ho *et al*. reported a 26.6% combined malignancy rate for all Bethesda category III nodules managed with surgery, repeat FNA, or observation, and a 37.8% malignancy rate for nodules managed by surgery alone.

The rate of categorization as persistent Bethesda category III on second FNA was 27.6%. Previous studies showed a wide range of 19.0–48.6% for classification as repeat Bethesda category III nodules on repeat FNA [4,9,13,14]. Rosario *et al*. reported 48.6% of repeat Bethesda category III nodules on a second FNA in their prospective repeat FNA study without surgery [9]. The reason for variable results among these studies may be due to different rates of repeat FNA versus surgery according to differing management guidelines for Bethesda category III nodules.

The risk of malignancy of repeat Bethesda category III nodules was 73.1% in our study, which was significantly higher than that of thyroid nodules classified initially as Bethesda category III. Bethesda category IV classification (follicular neoplasm or suspicious for follicular neoplasm) is thought to warrant surgery due to an estimated 15–30% risk of malignancy. Therefore, surgery should also be recommended for repeated Bethesda category III nodules based on our data. On the contrary, several previous studies have shown a lower risk of malignancy in repeat Bethesda category III nodules compared to those initially classified as Bethesda category III [4,15]. Broom *et al*. reported a malignancy rate that was slightly higher in the initial Bethesda category III group than the repeat Bethesda category III group (16.5% vs. 15.4%). As for nodules with confirmed surgical histopathology, however, the malignancy rate was lower in the initial Bethesda category III group than in the repeat Bethesda category III group (9.2% vs. 12.2%). Thus, further studies are necessary to determine the exact risk of malignancy and to generate consensus guidelines for management of repeat Bethesda category III nodules.

It is well established that certain ultrasonographic features are associated with malignancy in thyroid nodules [16,17]. Many previous reports have also emphasized the importance of those features in the management of Bethesda category III nodules [5,9,12,18]. Our results show that solid consistency, irregular/microlobulated margins, nonparallel shape, and “suspicious malignant” final US assessment were significantly associated with malignancy. On multivariate analysis, irregular/microlobulated margins was a significant independent factor associated with malignancy in repeat Bethesda category III nodules. Therefore, referring patients for surgical management on repeat Bethesda category III nodules with irregular/microlobulated margin on US may help to identify those at a higher risk of malignancy.

In this study, nodules that underwent direct surgery after initial Bethesda category III result had higher malignancy rate than nodules with repeat FNA (p < 0.05). However, direct surgery group also had higher probability of concurrent cancer, positive BRAF mutation and underlying thyroiditis (p < 0.05). These risk factors might let the surgeon decide surgery rather than repeat FNA and higher malignancy rate in direct surgery group can be explained.

In our study, clinically observed nodules unchanged or disappeared during the follow up within 1 year were considered as benign nodule in this study. Several previous studies reported malignancy rate of nodules to be classified as Bethesda category II on second FNA with a 3.5% (4/114) to 3.7% (2/54) of malignancy rate [3,9]. These malignancy rates are similar to that for patients diagnosed as benign according to the Bethesda system for thyroid cytopathology (assumed malignancy rate in the 0–3% range) for whom clinical follow-up is typically recommended as the usual management [1]. Although 6 of the 11 surgically resected lesions which were Bethesda II on second FNA on follow-up were malignant, those nodules should have been considered operation rather than second FNA which already had highly suspicious ultrasound features.

Because this was an observational retrospective study, it had several limitations. First, there may have been a selection bias since 377 nodules (48.8%) initially diagnosed as Bethesda category III were excluded due to lack of follow-up data. Second whether a surgical approach or repeat FNA was chosen depended on the preference of the clinician and the patient. Many Bethesda category III nodules are observed with no pathologic confirmation available and the indication for surgery is different across the institution. Differences may also relate to random variation or institutional differences in pathologic interpretation. These selection bias and referral bias could have resulted in an increase in the prevalence of malignancy in the Bethesda category III nodules in this study. Third, the number of patients classified as Bethesda category III three times was few, and whether malignancy risk increases with the number of repeat classifications could not be evaluated.

In conclusion, our study demonstrates that repeat Bethesda category III thyroid nodules on second FNA with irregular/microlobulated margins on US have an increased risk of malignancy, and that surgical management rather than repeat FNA or observation should be considered.
